# Renoprotective potential of exogen erythropoietin on experimental ruptured abdominal aortic aneurysm model: An animal study

**DOI:** 10.22038/IJBMS.2019.36215.8626

**Published:** 2020-02

**Authors:** Gokalp Altun, Yavuz Cakiroglu, Zerrin Pulathan, Esin Yulug, Ahmet Mentese

**Affiliations:** 1 Department of Cardiovascular Surgery, Faculty of Medicine, Karadeniz Technical University, 61080 Trabzon, Turkey; 2 Department of Histology and Embryology, Faculty of Medicine, Karadeniz Technical University, 61080 Trabzon, Turkey; 3 Department of Biochemistry, Faculty of Medicine, Karadeniz Technical University, 61080 Trabzon, Turkey

**Keywords:** Abdominal, Aneurysm, Aortic aneurysm, Erythropoietin, Hypovolemic, Ischemia-reperfusion injury, Renoprotective effect, Ruptured, Shock

## Abstract

**Objective(s):**

The aim of this study is to investigate the renoprotective effect of erythropoietin (EPO) on hypovolemic shock and ischemia/reperfusion (IR) injury on kidneys as end-organs in an experimentally-created ruptured abdominal aortic aneurysm (rAAA) model.

**Materials and Methods:**

Thirty anesthetized Sprague-Dawley male rats were randomized to sham ((Sh n:6) (Sh+EPO n:6)) or shock and I/R groups ((S/IR n:9) (S/IR+EPO n:9)). Additional surgical procedure except aortic exploration was not performed on Sh and Sh+EPO groups. 60 min of shock, 60 min of ischemia, and 120 min of reperfusion were applied on S/IR and S/IR+EPO groups. In the S/IR and S/IR+EPO groups, hemorrhagic shock, lower torso ischemia, and reperfusion were created. At the end of the shock period, saline solutions were separately and equally administered to Sh and S/IR groups, whereas 2000 U/kg EPO was intraperitoneally administered to Sh+EPO and S/IR+EPO groups. At the end of the experimental study, some biochemical and histological parameters were studied in serum and kidney tissues.

**Results:**

Biochemical parameters were all significantly increased in the S/IR group compared with the Sh group. These parameters were not statistically significantly different between S/IR+EPO and Sh+EPO groups. In histopathologic examination, EPO prevented high-grade injury.

**Conclusion:**

Our data indicate that EPO may have a renoprotective effect and reduce the systemic inflammatory response that resulted from shock and I/R in an experimental model of rAAA.

## Introduction

Ruptured abdominal aortic aneurysm (rAAA) is an important clinical pathology that is responsible for 1–2% of deaths of over 65-year-olds in society and is fatal when untreated. Although an increasing number of endovascular treatments (EVAR) has been performed for rAAA, nowadays, the most common treatment method in this condition is emergent surgical repair due to clinical or anatomic causes or difficulties related to team or equipment in the vascular center (1). 

Surgical treatment mortality of ruptured aneurysm is still above 40%. The most important causes of high mortality are hemorrhagic shock along with systemic organ injury in the preoperative period and addition of ischemia and reperfusion injury after surgical treatment (2, 3).

This condition causes multiple organ failure (MOF), which is the most important cause of mortality and morbidity in the postoperative period by causing local and end-organ injury in organs like the lungs, kidneys, heart, and liver. The most important cause of ischemia-reperfusion injury (IR) accompanying shock is systemic inflammatory response; therefore, drugs or treatment methods that may reduce mortality and morbidity are the ones for reducing the systemic inflammatory response (3).

The experimental model used in our study has been developed by Lindsay *et al.* and used in various studies. In this method, it has been shown that supramesenteric aorta clamp or shock are not the sole reasons for injury; and they cause significant systemic inflammatory response and end-organ injury when performed together like in rAAA (4). This rAAA model is a unique model that includes both hypovolemic shock and IR.

Erythropoietin (EPO) is actually a hematopoietic hormone. Its primary production zone is the kidneys in adults and the liver in fetuses. The most powerful stimulus for EPO is anemia. Hypoxia, inflammation, and cell death are other causes of its increased production. It has been shown in various studies that increased expression of EPO has strong tissue-protective effects (antiapoptotic, antioxidant, angiogenic, and neuroprotective) against IR injury in addition to its hematopoietic effect (5-8).

This study aims to demonstrate the protective effect of EPO on kidneys in rAAA models that involve both shock and IR injury.

## Materials and Methods

***Animal Care***

In this study, a total of 30 Sprague-Dawley type male rats weighing 420±60 g were used after obtaining approval of Karadeniz Technical University Animal Experiments Local Ethics Committee (approval no. 2010/5-1). Subjects were kept at 23 ^°^C room temperature and 12 hr dark/light cycle with standard bait and water, then they were given only water for 12 hr at night before the experiment. Care of rats was done in accordance with the Guide for the Care and Use of Laboratory Animals (NIH press no. 85-23, 1985 revised).

***Experimental design***

Rats were sedated with intraperitoneal ketamine (50 mg/kg) and Xylazine (10 mg/kg). Anesthesia was sustained by preserved spontaneous respiration with intermittent ketamine administration. Subjects were randomized into two groups as Sham (Sh) and Shock/IR(S/IR) groups, then a total of 4 groups were created by dividing every group to two more groups, and operator was blind to these assignments: Sh (n=6), Sh+EPO (n=6), S/IR (n=9), and S/IR+EPO (n=9). Additional surgical procedure except aortic exploration was not performed on Sh and Sh+EPO groups. 60 min of shock, 60 min of ischemia, and 120 min of reperfusion were applied to S/IR and S/IR+EPO groups (Table 1). 

Heart rate, mean arterial pressure (MAP) (by right jugular vein and right carotid artery cannulations), respiratory rate, and rectal temperature of rats were monitored after anesthesia (Nikon Konden BSM-4113). 3 ml/kg/hr of saline (0.9% NaCI) infusion was performed (Perfusor CompactS, Brown), and rectal temperature was kept at 36.5 ^°^C with a heat lamp during the whole experiment. Aneurysm rupture was simulated by creating shock withdrawing blood from the carotid artery into a heparinized injector as MAP was kept at 50 mmHg for 60 min in S/IR groups, then withdrawn blood was kept at the room temperature. At the end of 60 min of the shock period, saline solutions were separately and equally administered to Sh and S/IR groups whereas 2000 U/kg EPO (EPREX© 2000 IU/ml, Janssen-Cilag AG, Santa Farma, Istanbul, Turkey) was intraperitoneally (IP) administered to Sh+EPO and S/IR+EPO groups. The abdominal aorta was explored with midline laparotomy. All groups received 250 U/kg of IV systemic standard heparin; then, lower body ischemia was created by clamping the abdominal aorta at proximals of superior mesenteric artery and iliac bifurcation with microvascular clamps in S/R and S/IR+EPO groups. Laparotomy was applied to Sh and Sh+EPO groups and no other procedures (i.e. Shock and Ischemia/Reperfusion) were performed until the end of the experiment after approximating laparotomy incisions with sutures. Surgical x-clamp and resuscitation were simulated in S/IR groups by administrating half of the previously withdrawn blood through the venous line after placing the aorta clamps. After 60 min of ischemic period, all of remaining withdrawn blood was reinfused just before opening the clamp, and then clamps were opened. The abdomen was closed again, and rats were left to reperfuse for 120 min. MAP was kept at 100 mmHg during reperfusion by administrating additional saline solution if necessary. Euthanasia was performed at the end of the experiment by drawing blood from the carotid artery. Both kidneys were removed; the extracted kidneys were cut carefully into two parts. Half of the tissues were frozen and kept at -80 ^°^C for biochemical analyses, whereas the other halves were fixated in formaldehyde for histopathological examinations. 

***Laboratory analysis***

Serum malondialdehyde (MDA), myeloperoxidase (MPO), tumor necrosis factor-alpha (TNF-α), blood urea nitrogen (BUN), and creatinine (Cre) levels were measured in blood samples. MDA and MPO in kidney tissue were measured, and histopathologic examination was performed.

***Serum MDA measurement***

MDA levels in rat plasma samples were measured by using TBARS (Thiobarbituric Acid Reactive Substance) method which was developed by Yagi in 1984. Red color occurred by reaction between MDA, which is a lipid peroxidation product, and thiobarbituric acids were spectrofotometrically measured at 532 nm wavelength. MDA levels were calculated as nmol/ml as result of measurement (9).

***Tissue MDA measurement***

A piece of kidney tissue was used to measure MDA levels. The sample was minced and homogenized in an ice-cold 1.15% KCl solution containing 0.05% Triton X-100 with the use of an Ultra-Turrax T25 homogenizer (Janke and Kunkel IKA, Staufen, Germany). Tissue MDA levels were determined with use of the method described by Mihara and Uchiyama (10). Tetramethoxypropane was used as a standard, and MDA levels were calculated as nmol/g of wet tissue.

***Serum MPO measurement***

MPO levels in rat plasma samples were determined by using an enzyme-linked immunosorbent assay (ELISA) kit (Hycult biotech, Catalog No. HK105, The Netherlands) according to the manufacturer’s instructions. The absorbance of samples was measured by the VERSA microplate reader (Designed by molecular Devices in California, USA) at 450 nm wavelength. Results were given as ng/ml.

***Tissue MPO measurement***

MPO levels in tissues were determined by using an enzyme-linked immunosorbent assay (ELISA) kit (Hycult biotech, catalog no. HK105, The Netherlands) according to the manufacturer’s instructions. The absorbance of samples was measured using a VERSA microplate reader (Designed by molecular Devices in California, USA) at 450 nm wavelength. Results were given as ng/ml/g of tissue unit.

***Serum TNF-*** ***α*** ***measurement***

SerumTNFα levels were measured by using an enzyme-linked immunosorbent assay (ELISA) kit (Bender MedSystems, catalog no. BMS622, Vienna, Austria). Sample absorbance was measured in a VERSA microplate reader (Designed by molecular Devices in California, USA) at 450 nm wavelength. Results were given in pg/ml.

***Serum BUN and Creatinine Measurements***

Serum BUN and Cre levels were measured in the Roche/Cobas 6000 system, using original reagents.

***Histopathologic evaluation of kidney tissue***

After the termination of the experiment, kidney tissues were separately placed in numbered containers involving 10% neutral formaldehyde solution. Solutions polluted with blood were replaced after 30 min, and tissues were fixated in 10% neutral formaldehyde solution for 48 hr. They were passed through alcohol series for dehydration, and then they were embedded in paraffin block. Prepared paraffin blocks were cut to 5 μm thickness with a microtome (Leica RM2255, Japan), then stained with hematoxylin-eosin (H&E). Histopathologic evaluation was performed by an experienced histologist who was unaware of study groups. All of the kidney tissue preparations were observed and photographed with a digital camera (Olympus DP71, Olympus, Tokyo, Japan) attached to a photomicroscope (Olympus BX51, Olympus, Tokyo, Japan). In injury scoring of kidney tissues, tissues of every group were semi-quantitatively evaluated in high magnification (400x) with 5 different areas under a light microscope. Each section was scored semi-quantitatively from 0-3 (0: no damage; 1: mild=<25% damage, focal, slight changes; 2: moderate= 25–50% damage, multifocal, significant changes and 3: severe=>50% damage, common widespread changes) according to defined criteria: focal glomerular necrosis, Bowman capsule dilatation, degeneration of tubular cells, and intertubular vascular congestion. Tubular dilatation, shedding in tubular epithelial cells, and vacuolization in tubular cells were evaluated for tubular degeneration. In scoring, cortex and outer medullary areas were focused for injury evaluation. 

***Statistical analysis***

Statistical analysis was performed using SPSS (ver. 24.0). Kruskal-Wallis variance analysis (*post hoc* test was performed to test the significance of pairwise differences using Bonferroni correction to adjust for multiple comparisons) was used to compare the groups. The results were expressed as mean+standard deviation (SD). Statistical significance was set at *P*<0.05.

## Results

***Serum MDA***

In paired comparisons of groups, serum MDA value was found to be significantly higher in S/IR group compared with the Sh group (*P*=0.002); however, it was not considered as statistically significant (*P*=0.019) in S/IR+EPO group although it fell a little in this group. There was no difference between Sham and Sh+EPO groups in terms of MDA values (*P*=1.0).

***Serum MPO***

In paired comparisons of groups, serum MPO value was significantly increased (*P*=0.001) in S/IR group compared with the Sh group, but there was no statistically significant difference between Sh and Sh+EPO groups. However, there was an increase in S/IR+EPO compared with the Sham group, whereas there was a decrease in S/IR+EPO group compared with the S/IR group, and it was statistically significant (*P*<0.01).

***TNF-α***

In paired comparisons of groups, serum TNF-α value was significantly higher (*P*=0.001) in S/IR group compared with the Sh group. Values were decreased in Sh+EPO; however, it was similar to the levels of the Sh group, and therefore it was not statistically significant. There was an increase in S/IR+EPO group compared with the Sh group, whereas there was a decrease in S/IR+EPO group compared with the S/IR group, which was statistically significant (*P*<0.01).

***Tissue MDA***

In paired comparisons of groups, tissue MDA value was significantly increased in S/IR group compared with the Sh group (*P*=0.001), and it was statistically significant. Values were decreased in Sh+EPO group like Sh group values; however, it was not statistically significant (*P*=0.937). There was an increase in S/IR+EPO group compared with the Sh group, whereas there was a decrease in S/IR+EPO group compared with the S/IR group, and it was statistically significant (*P*<0.01).

***Tissue MPO***

In paired comparisons of groups, tissue MPO value was significantly higher in S/IR group compared with the Sh group, and it was statistically significant (*P*=0.001). Values were decreased in Sh+EPO group like Sh group values; however, it was not statistically significant (*P*=1.000). There was an increase in S/IR+EPO group compared with the Sh group, whereas there was a decrease in S/IR+EPO group compared with the S/IR group (*P*=0.019); however, it was not statistically significant (*P*<0.01).

***BUN***

In paired comparisons of groups, the BUN value was higher in S/IR group compared with the Sh group; however, it was not statistically significant (*P*=0.222). Sh+EPO group’s values were quite decreased to even below the levels of Sh group; however, it was not statistically significant (*P*=0.180). There was a decrease in S/IR + EPO group compared with Sh and S/IR groups (*P*=0.222); however, it was not statistically significant (*P*<0.01).

***CRE***

In paired comparisons of groups, the CRE value was increased in S/IR group compared with the Sh group (*P*=0.094), and it was not statistically significant. There was a prominent decrease in Sh+EPO group values like Sh group values (*P*=0.818); however, it was not statistically significant. There was an increase in S/IR+EPO group compared with the Sh group, whereas there was a decrease in S/IR+EPO group compared with the S/IR group (*P*=0.094); however, it was not statistically significant (*P*<0.01) (Table 2).

***Histopathologic findings***

**Figure 1:**
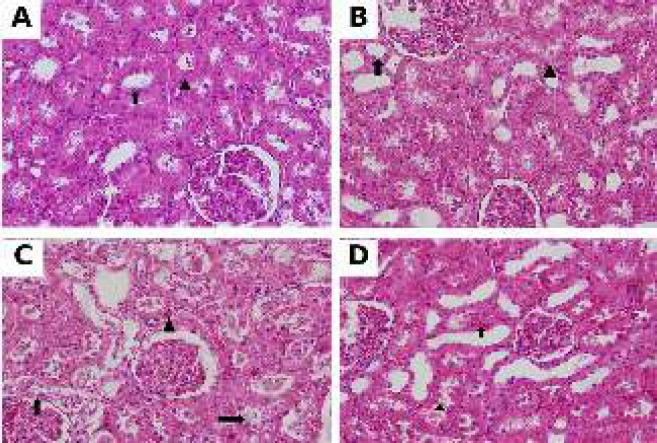
(A) Histopathological examination of sham group revealed normal architecture

Normal kidney structure was observed on preparations of the Sh group. Proximal and distal tubules appeared normal. Normal kidney histologic structure like in the Sh group was observed in kidney tissue preparations of Sh+EPO group. Prevalent vacuolization, shedding of epithelial cells into tubule lumen, and extensive vascular congestion in intertubular areas of proximal and distal tubule epithelium cells were observed in preparations of Sh+I/R group. Mild tubular cell degeneration and mild intertubular congestion were observed in S/IR + EPO group (Figure 1).

According to the chi-square test, tubular epithelial degeneration was predominantly Grade 0 (66.7%) and Grade 1 in Sh group, whereas it was most frequently (66%) Grade 3 in S/IR group. Damage at 66% in S/IR group was found to be reduced to 11.1% in S/IR + EPO group, which had received EPO. However, only Grade 2 damage with a percentage of 16.7% was found to be different from the Sh group.

According to the chi-square test of kidney intertubular vascular congestion grading, Grade 2 damage (66.7%) was most frequent in S/IR group although Grade 0 and Grade 1 were most prevalent in the Sh group. This rate fell to 55.6% in the group receiving EPO. Also, Grade 3 damage was observed with a rate of 1.1% in S/IR group, whereas no Grade 3 damage was observed in S/IR+EPO group. As different from the Sh group, Grade 2 damage with a rate of 50% was observed in Sh group receiving EPO (Table 3).

## Discussion

In this study, the protective effect of EPO on hypovolemic shock and I/R injury on kidneys as an end-organ in an experimentally-created rAAA model was investigated. Serum MDA, serum MPO, tissue MDA, tissue MPO, TNF-α, BUN, and creatinine were all significantly increased in S/IR group compared with the Sh group. Serum MPO, tissue MDA, tissue MPO, and TNF-α levels were not significantly different between S/IR+EPO and Sh+EPO groups. There was no statistically significant difference between control and study groups in terms of BUN and creatinine levels. These findings indicate that EPO is predominantly effective in reducing I/R injury, which especially occurred with shock. It was observed that intraperitoneally administered EPO was effective in reducing ischemic damage caused by free oxygen radicals. It was also observed that EPO reduces cellular damage by suppressing neutrophil activation during reperfusion stage.

In histopathologic examination, tubular and intertubular damage was scored and grading was performed. Grade 0 and Grade 1 damage were most frequent in the Sh group, whereas Grade 2 and Grade 3 damage were most frequent in the S/IR group. It was observed that Grade 3 damage with a rate of 66% was reduced to 11% in the group receiving EPO. Therefore, we can say that EPO had a preventing effect on tubular damage. When compared with Sh and Sh+EPO groups, there was also a little increase in grade in group receiving EPO (Grade 1 damage in Sham group was increased from 33% to 83%). This indicates that EPO itself caused some degree of damage that may be related to dosage. When evaluating intertubular damage, it was observed that EPO reduced Grade 3 damage with a rate of 11% and Grade 2 damage with a rate of 55% in S/IR group to 0% and 55% in S/IR+EPO group, respectively. Also, Grade 2 damage with a rate of 0% in Sh group was increased to 50% in Sh+EPO group. EPO prevented high-grade injury; however, it increased Grade 1 level injury.

EPO is a glycoprotein with a molecular weight of 30.4 kDal. Its primary production area is kidney in adults and liver in fetuses (7,8). The most potent stimulator of its production is anemia. Its release rate increases during bleeding conditions with reduced erythrocyte production and hypoxia. The specific effect of EPO is stimulating differentiation and proliferation of erythroid progenitor cells as well as supporting the survival capability of erythroid cells during the maturation period (11-13). In addition, many protective effects of EPO, such as anti-apoptotic, antioxidant, angiogenic, and neuroprotective effects against ischemia have been demonstrated in cell cultures and animal models (14-16).

EPO, which has been described as a renal hormone causing erythrocyte production, has been known to locally increase as a response to physical and metabolic stress in many tissues. Kidneys are the primary source of EPO production. EPO production occurs as a response to hypoxia and does not require other plasma factors for synthesis (7). It causes pre-conditioning (ischemic tolerance) with its autocrine and paracrine roles, and it restricts destructive potentials of proinflammatory cytokines on brain, heart, kidney, and other tissues (12). Due to suppression of local EPO production due to ischemic damage, exogen EPO administration has become a successful treatment approach against renal injury caused by I/R toxicity in clinical and pre-clinical studies (11-13). The main mechanisms of EPO on renoprotection can be stated as preventing toxicity caused by glutamate, inhibition of apoptosis, anti-inflammatory effects, and stimulation of angiogenesis (15,16). 

In light of these findings and results of previous studies, EPO may reduce tissue damage at the injury zone by non-hemopoietic receptors. EPO also prevents apoptosis in presence of reduced oxygenation, excitotoxicity, and increased free radicals. EPO may reduce kidney injury caused by ischemia/reperfusion, and it may facilitate kidney tubular regeneration by reducing severity of kidney failure (14-16). EPO may have damage-reducing effect in end-organs in conditions with co-influence of shock and ischemia/reperfusion like RAAA.

**Table 1:** Schematic diagram of the experimental design

Sh		Aortic exploration+ Saline infusion	
Sh+EPO		Aortic exploration+EPO infusion	
S/IR	Shock 60 min	Ischemia 60 min+Saline inf.	Reperfusion 120 min
s/IR+EPO	Shock 60 min	Ischemia 60 min+EPO inf.	Reperfusion 120 min

Sh: Sham group (n=6); Sh+EPO: Sham+Erythropoietin group (n=6); S/IR: Shock+Ischemia/Reperfusion group (n=9); S/IR+EPO: Shock+Ischemia/Reperfusion+Erythropoietin group (n=9)

**Table 2:** Biochemical parameters of all groups (mean±SD). The results were expressed as mean+standard deviation (SD)

**Parameters**	Sh (n = 6)	Sh+EPO (n = 6)	S/IR (n = 9)	S/IR+EPO (n = 9)
MPO (ng/mL)	81.83 ± 52.25	83.34 ±17.02	383.85 ±38.01^a,b^	188.82 ±10.47^a,b,c^
MDA (nmol/mL)	1.46 ±0.48	1.56 ± 0.20	2.95 ±1.48^a,b^	1.88 ±0.51^a,b,c^
TNF𝛼 (pg/mL)	151.63 ±30.64	159.08 ±24.12	262.71 ±18.24^a,b^	206.99 ±14.92^a,b,c^
Tissue MPO (nmol/g)	1177.10 ±89.33	2136.50 ±1313.82	5419.15 ±646.69^a,b^	4365.00 ±743.33^a,b,c^
Tissue MDA (ng/mL)	2153.40 ±1389.79	1197.97 ± 361.84	2479.32 ± 637.15^a,b^	1662.22 ± 343.78^a,b,c^
BUN (mg/dl)	456.66 ±101.72	344.00 ±140.94	475.00 ±161.76^a,b^	417.11 ±86.66^a,b,c^
CRE (mg/dl)	23.00 ±3.03	23.83 ±6.82	37.22 ±3.19^a,b^	34.77 ±4.14^a,b,c^

Sh: sham; EPO: Erythropoietin; S/IR: Shock+Ischemia/Reperfusion; MDA: malondialdehyde; MPO: myeloperoxidase; TNF-α: tumor necrosis factor alpha; BUN: Blood Urea Nitrogen; Cre: Creatinine. ^a ^*P<*0.016 versus Sh; ^b ^*P<*0.016 versus Sh+EP0; ^c^ *P<*0.016 versus S/IR

Proximal tubule (▲); Distal tubule (↑). (B) Histoarchitecture of Sham+EPO group appeared normal. Proximal tubule (▲); Distal tubule (↑). (C) Histopathological view of the Shock+I/R group. Tubular epithelial cells showed extensive vacuolization, lumen spilled epithelial cells (↑), and extensive intertubular vascular congestion (▲). (D) Histopathological view of Shock+I/R+EPO group. Tubule epithelial cells were near-normal. Mild degeneration of epithelial cells (▲) and mild increase of intertubular vascular congestion (↑) were noted (Hematoxylin-Eosin: H&E, X200)

**Table 3:** Histological grading of degeneration of tubular epithelial cells (*) and intertubular vascular congestion (✦) of all groups

	Grade 0		Grade 1		Grade 2		Grade 3	
	*	✦	*	✦	*	✦	*	✦
Sham	%66,7	50%	%33,3	%50,0	0	0	0	0
S+I/R	0	0	0	%22,2	%33,0	%66,7	%66,0	%11,1
Sham+EPO	0	0	%83,3	%50,0	%16,7	%50,0	0	0
S+I/R+EPO	0	0	%11,1	%44,4	%77,8	%55,6	%11,1	0

## Conclusion

The protective effect of EPO on hypovolemic shock and I/R injury in kidneys as end-organs in an experimentally-created rAAA model was investigated in this study. The Significant increase in kidney tissue MDA values of the I/R group may be an indicator of oxidative stress in kidney tissue. Histopathologic changes of kidney tissues in the I/R group may be a finding of activation of neutrophil chemotaxis and adhesion apart from the organ with ischemia. During our study, it was found that shock and lower extremity I/R injury cause kidney end-organ injury. Also, significant reduction in MDA and MPO values in S/IR+EPO group indicates reduced oxidative stress and inhibited neutrophil-induced systemic inflammatory response. In addition, milder histopathologic changes in EPO receiving group compared with the I/R group may be due to inhibition of neutrophil chemotaxis and adhesion by EPO.
